# Biotechnology, Bioengineering and Applications of *Bacillus* Nattokinase

**DOI:** 10.3390/biom12070980

**Published:** 2022-07-13

**Authors:** Li Yuan, Chen Liangqi, Tang Xiyu, Li Jinyao

**Affiliations:** 1 Department of Materia Medica, Xinjiang University, Urumqi 830017, China; liyuanstc@xju.edu.cn; 2 Xinjiang Key Laboratory of Biological Resources and Genetic Engineering, College of Life Science and Technology, Xinjiang University, Urumqi 830017, China; clqxju@stu.xju.edu.cn (C.L.); txyxju@stu.xju.edu.cn (T.X.)

**Keywords:** *Bacillus* nattokinase, physiological and biochemical characteristics, molecular structure, molecular modification, functional food and clinical application

## Abstract

Thrombosis has threatened human health in past decades. *Bacillus* nattokinase is a potential low-cost thrombolytic drug without side-effects and has been introduced into the consumer market as a functional food or dietary supplement. This review firstly summarizes the biodiversity of sources and the fermentation process of nattokinase, and systematically elucidates the structure, catalytic mechanism and enzymatic properties of nattokinase. In view of the problems of low fermentation yield, insufficient activity and stability of nattokinase, this review discusses the heterologous expression of nattokinase in different microbial hosts and summarizes the protein and genetic engineering progress of nattokinase-producing strains. Finally, this review summarizes the clinical applications of nattokinase.

## 1. Nattokinase Is a New Type of Thrombolytic Drug with Great Potential

The incidence of cardiovascular diseases has increased significantly worldwide and showed a trend of younger onset. Among cardiovascular diseases, cerebral infarction, ischemic stroke, and myocardial infarction are all related to thrombi formed by the coagulation of fibrin and platelets, and the current clinical application of thrombolytic agents including urokinase, tissue plasminogen activator (t-PA) and streptokinase all have serious side effects such as bleeding or gastric ulcer [1,2]. Therefore, the search for effective and safe thrombolytic drugs has become one of the directions in the field of cardiovascular disease research.

Natto, which is fermented by inoculating soybeans with *Bacillus subtilis*, is a traditional food with a long history in Japan. Intake of natto and other related fermented soy products is inversely related to the incidence of cardiovascular diseases, hence long-term consumption of natto is considered to be one of the important reasons for the longevity of Japanese [3]. Nattokinase is a kind of alkaline serine protease with strong fibrinolytic and thrombolytic activity, which is secreted by *Bacillus* natto and discovered in natto by Sumi et al. [4]. Compared with traditional thrombolytic drugs, nattokinase has a relatively lower risk of delivery, a larger tolerable dose, and lacks side effects such as gene mutation and chromosomal aberration induction [5,6]. More importantly, nattokinase also has various pharmacological effects, such as improving microcirculation and lowering blood pressure [7], anticoagulation [8], preventing atherosclerosis [9], relieving retinal angiogenesis [10], anticancer [11], inhibiting inflammation and oxidative stress, etc. [12]. In conclusion, nattokinase is a new type of thrombolytic drug with great application potential [13].

In recent years, the research on nattokinase in various fields has progressed rapidly. This review aims at offering an up-to-date comprehensive summary of *Bacillus* nattokinase. We firstly addressed the diversity of synthetic nattokinase strains, optimization of nattokinase fermentation conditions, and then enzyme structure and catalytic mechanism, enzymatic properties, molecular modification, heterologous expression, and finally, the latest progress in the application of nattokinase with a systematic discussion.

## 2. *Bacillus* Is the Main Strain for Synthesizing Nattokinase

Nattokinase is mainly produced by fermentation of *Bacillus*. Some marine organisms [14] and *Pseudomonas* sp. [15] also produce nattokinase, such as *Pseudomonas aeruginosa* CMSS [15] screened from cow’s milk and *Pseudomonas* sp. [16] obtained from the soil. Although nattokinase was first isolated from the Japanese food natto, similar fibrinolytic enzyme-producing strains are also available from other traditionally fermented foods: *Bacillus subtilis* Natto B-12 [17] and *Bacillus subtilis* JNFE0126 [18] were isolated from natto; *Bacillus amyloliquefaciens* DC-4 [19], *Bacillus subtilis* LD-8547 [20] and *Bacillus sublitis* DC33 [21] were obtained from Chinese traditional fermented food tempeh; *Bacillus subtilis* LSSE-62 [22] was obtained from Chinese soybean paste; and *Bacillus* sp. strain CK 11-4 [23] and *Bacillus subtilis* WRL101 [24] were isolated from Chungkook-Jang, a traditional Korean fermented food. In addition to fermented food sources, *Bacillus cereus* VITSDVM3 [25], isolated from rust, was also confirmed as a potent nattokinase producer (Table 1). Overall, most of the current research objects are mainly nattokinase derived from *Bacillus subtilis* natto screened in Japanese natto.

**Table 1:** Biodiverse sources of nattokinase.

Strain	Source	References
*Bacillus subtilis* Natto B-12	Natto	[17]
*Bacillus* sp. *strain* CK 11-4	Chungkook-Jang	[23]
*Bacillus* sp. *strain* DJ-4	Doen-Jang	[26]
*Bacillus amyloliquefaciens* DC-4	Douchi	[19]
*Bacillus subtilis* DC33	Douchi	[21]
*Bacillus subtilis* QK02	Fermented soybeans	[27]
*Bacillus subtilis* LD-8547	Douchi	[20]
*Bacillus subtilis* RJAS19	Fermented soy products	[28]
*Bacillus subtilis* YJ1	Fermented soy products	[29]
*Bacillus subtilis* TKU007	Soil	[30]
*Bacillus sublitis*	Thailand	[31]
*Bacillus subtilis* LSSE-22	Chinese soybean paste	[32]
*Pseudomonas* sp. TKU015	Soil	[16]
*Pseudomonas aeruginosa* CMSS	Milk	[15]
*Bacilluscereus* VITSDVM3	Rust	[25]
*Bacillus subtilis WRL101*	Doen-jang	[24]
*Bacillus velezensis* KMU01	Pickle	[33]
*Bacillus subtilis* VITMS 2	Fermented milk of *Vigna unguiculata*.	[34]
*Bacillussubtilis* JNFE0126	Natto	[18]
*Bacillus subtilis* K2	Moromi	[35]
*Bacillussubtilis* LSSE-62	Chinese soybean paste	[22]
*Bacillus subtilis* ICTF-1	Ocean	[36]

As a probiotic, *Bacillus*, which can synthesize nattokinase, has great potential in the fields of functional food and pharmaceutical applications. However, qualified oral nattokinase probiotics need to have the ability to overcome the special environment (gastric acid, bile salts, protease, etc.) of the human digestive system. However, none of the strains discovered so far have had their acid resistance and bile salt resistance reported. Therefore, the development of nattokinase synthetic probiotics adapted to the human digestive system has become one of the future research directions.

## 3. Structure and Catalytic Mechanism of Nattokinase

### 3.1. Nattokinase: The Only Member of the Alkaline Serine Protease Family with Thrombolytic Activity

**Figure 1:**
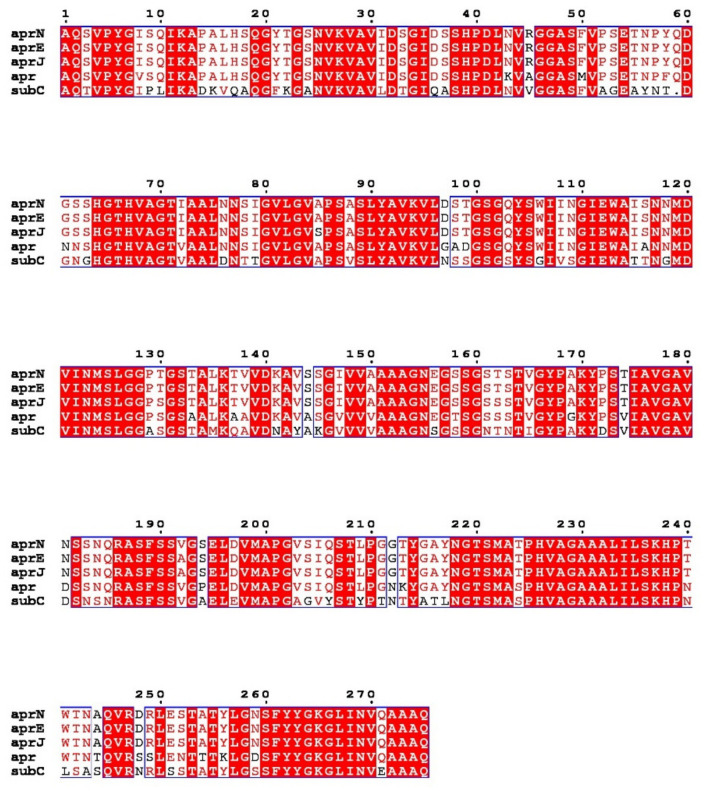
Multiple sequence alignment of nattokinase (*Apr*N) compared with other serine protease homologs. Red shading and red words represent identical and similar residues, respectively. *Apr*E: Subtilisin E; *Apr*J: Subtilisin BPN′; *Apr*: Subtilisin Carlsberg.

Nattokinase (3.4.21.62) belongs to the family of alkaline serine proteases. As an endogenous fibrinolytic enzyme, nattokinase is functionally similar to human plasmin (3.4.21.7; 75 kDa). In 1992, Nakamura et al. used the shotgun method to determine that the gene encoding nattokinase (*apr*N) starts from GTG, has an open reading frame of 1146 bp, and encodes 381 amino acids, including a signal peptide of 29 amino acids, a propeptide of 77 amino acids, and a mature peptide of 275 amino acids with a molecular weight of 27.7 kDa. Since nattokinase is a cysteine-free protease, no disulfide bonds are observed in its structure. The open reading frame of nattokinase contains three consecutive terminators (TAATAGTAA) and is regulated by Rho-independent factors [37]. In silico analysis showed that nattokinase had 99.5%, 86%, and 72% sequence homology with subtilisin E, subtilisin BPN′, and subtilisin Carlsberg, which belong to the same alkaline serine protease family. The three amino acid residues (Ser221, His64 and Asp32) necessary for the catalytic center of serine proteases and the region near the catalytic triad are highly conserved among the above alkaline serine protease family members (Figure 1) [38]. Although nattokinase is highly homologous to many subtilisins in the serine protease family, only a few proteins, such as nattokinase, show high substrate specificity to fibrin and can directly cleave cross-linked fibrin in vitro and in vivo [39].

### 3.2. Structure and Reaction Mechanism of Nattokinase

**Figure 2:**
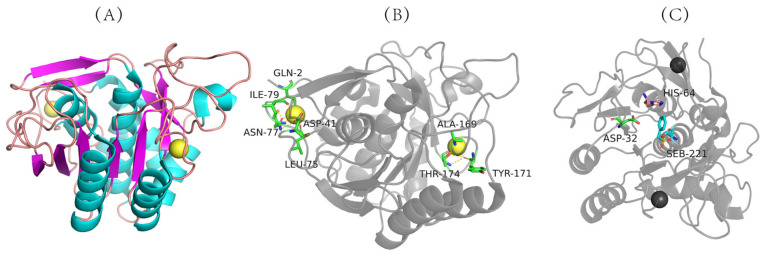
Three-dimensional structure of nattokinase. (**A**) Calcium binding site of nattokinase. (**B**) Three-dimensional structure of nattokinase. (**C**) Nattokinase triple catalyst. Nattokinase structure diagram taken from https://www.rcsb.org/structure/4DWW with modifications (accessed on 14 March 2012).

The three-dimensional structure of nattokinase derived from *Bacillus natto* has been successfully analyzed (PDB code: 4DWW (2022) https://www.rcsb.org/structure/4DWW (accessed on 14 June 2022)) (Figure 2A), which shows that nattokinase is a single-chain polypeptide without disulfide bonds. Mature peptides consist of 9 α-helixes, 9 β-sheets and 2 Ca^2+^ binding sites (Gln2, Asp41, Leu75, Asn77, Ile79, Val81, Ala169, Tyr171, Thr174) for structural stability (Figure 2A,B). The catalytically active center of nattokinase consists of a conserved catalytic triad (Asp32, His64, Ser221), while its substrate-binding center contains three conserved amino acids (Ser125, Leu126, Gly127) (Figure 2B) [40]. Similarly to other subtilisin proteases, the seven typical β-sheets of nattokinase are located near the center of the enzyme molecule, and the other two β-sheets are inversely located in the domain near the C-terminus; the 9 β-sheets of nattokinase are assembled in reverse with the 9 α-helices, of which 7 α-helices are on the same surface [41].

The catalytic mechanism of nattokinase has not yet been reported. Since the three-dimensional structure of nattokinase highly overlaps with that of other alkaline serine proteases in its family, its molecular mechanism is similar to that of the alkaline serine protease family. First, the ring nitrogen atom of the His64 residue in the catalytically active center receives the hydroxyl proton of Ser221, which enhances the nucleophilic ability of Ser221 and attacks the hydroxyl carbon of the peptide bond of the substrate to form a tetrahedral transition state intermediate. Asp32 stabilizes the protonation state of His64 through the negative charge of the carboxyl group. Next, His64 donates a proton to the newly formed amino group to release the first product (acylation reaction) while forming a covalent acyl-enzyme complex. As the water molecule nucleophilically attacks the covalent acyl–enzyme complex to form a tetrahedral intermediate, His64 transfers the proton back to Ser221, and the transition state disintegrates to release the first product, thereby completing the deacylation reaction [40,41,42].

### 3.3. The Propeptide of Nattokinase Is Involved in the Correct Folding of Nattokinase as an Intramolecular Chaperone

The propeptide of nattokinase plays a key role in the correct folding of nattokinase. By comparing the thrombolytic activities of nattokinase holoenzyme (propeptide + mature peptide) and nattokinase mature peptide, Weng et al. found that only nattokinase expressing both propeptide and mature peptide has thrombolytic activity, inferring that the propeptide may be involved in the correct folding of nattokinase as an intramolecular chaperone [43]. Based on the structural similarity of serine protease family proteins, some studies have elucidated the role of the catalytic triplet in cleavage between the intramolecular chaperone and the nattokinase mature peptide. Asp32 assists in positioning the correct tautomer of His64, and Ser221 transfers its proton to His64 with increased nucleophilicity, which in turn completes substrate cleavage by nucleophilic attack on the carbonyl carbon of the propeptide’s peptide bond. However, the catalytic mechanism of this theory is being questioned [44].

The protein structure of nattokinase has now been resolved. However, the molecular mechanism of thrombolysis induced by nattokinase and the role of propeptide in the correct folding of nattokinase still need to be elucidated.

## 4. Study on Fermentation Process of Nattokinase

Obtaining nattokinase by strain fermentation is the main process for the industrial production of nattokinase. Therefore, it is of great significance to study the fermentation process of nattokinase to improve its fermentation yield. The traditional natto fermentation process involves wrapping steamed soybeans with boiled rice straw and fermenting at 40 °C for about one day. At present, the industrial fermentation modes for nattokinase are divided into solid fermentation and liquid fermentation, and the main goals of nattokinase fermentation technology research are to reduce the cost and improve the yield.

### 4.1. Production of Nattokinase by Solid Fermentation

The selection of raw materials is a key factor for the production of nattokinase by solid-state fermentation. In general, soybean, chickpea, and wheat bran are mainly used as raw materials. Some by-products produced in the process of synthesizing nattokinase by solid-state fermentation can improve the synthesis efficiency, but may also increase the difficulty of purification of fermentation products and lead to problems with drug safety. It has been reported that poly-L-glutamic acid (γ-PGA) is produced during the synthesis of nattokinase by solid-state fermentation. However, the effect of this by-product on the fermentation of nattokinase is still controversial. Li et al. found that the activity of nattokinase was the highest when the production of γ-PGA reached the maximum value; thus Li et al. speculated that the γ-PGA produced by *Bacillus subtilis* during the fermentation process could stimulate the biosynthesis of nattokinase [45]. Contrarily, Wei et al. believed that γ-PGA mixed in nattokinase was an immunogenic substance that would cause allergic reactions in humans that consume it. Therefore, Wei used chickpeas instead of soybean as the fermentation carbon source for the fermentation of nattokinase and found that the chickpea medium could effectively reduce the biosynthesis of γ-PGA [32]. By optimizing chickpea loading and fermentation temperature, the fermentation yield of nattokinase could be increased to 39.28 FU/g, indicating that the fermentation conditions (temperature, time and the concentration of substrates) could determine the yield of nattokinase [22].

In general, the cost and energy consumption of nattokinase solid-state fermentation are lower than that of liquid fermentation, and the technology is relatively simple, which is suitable for small-scale production. At present, there have been reports on the successful commercial application of nattokinase [46]. However, the solid-state fermentation culture method has obvious defects: (1) the process of solid-state fermentation is relatively extensive, and the intermediate parameters of the fermentation process are difficult to detect; (2) the solid-state fermentation medium has less water content and poor fluidity, which limits the yield of nattokinase; (3) it is difficult to purify the products obtained by solid-state fermentation [32]. Therefore, large-scale industrial production of nattokinase using solid-state fermentation needs to be further studied.

### 4.2. Production of Nattokinase by Liquid Fermentation

Nattokinase liquid fermentation mainly uses various monosaccharides and soluble starch as carbon sources and peptone and soybean meal as nitrogen sources. The related research on liquid fermentation of nattokinase so far is shown in Table 2. In general, different media and fermentation methods directly affect the yield and activity of nattokinase. For nattokinase liquid fermentation medium, glucose was considered to be an effective carbon source, while peptone, yeast extract and soybean peptone were proved to be suitable nitrogen sources for nattokinase production, among which soybean peptone, yeast extract, and calcium chloride had a significant effect on the yield of nattokinase. In recent research, inorganic salts also affected the fermentation yield of nattokinase: the addition of calcium ions to the fermentation system can affect the adhesion ability of *Bacillus subtilis* by participating in the synergistic effect with the enzymes responsible for anchoring on the cell wall; the appropriate concentration of magnesium ions in the fermentation medium can also enhance the synthesis of *Bacillus subtilis* peptidoglycan, thereby enhancing the strength of the cell wall to increase the yield of nattokinase [47]. There are also a few reports confirming that the addition of glycerol and selenium also helps to improve the yield of nattokinase liquid fermentation. Adding an appropriate amount of glycerol (6%) to the fermentation medium can effectively increase the cell density of *Bacillus subtilis* natto in the fermentation broth, which in turn helps to improve the fermentation yield of nattokinase [48]. Selenium (5 g/L) contained in nattokinase fermentation medium can improve not only the fermentation yield of nattokinase, but also its antioxidant activity and thermal stability, which confirms that selenium can improve the quality of nattokinase [49].

**Table 2:** Liquid fermentation conditions for nattokinase.

Strain	Carbon source	Nitrogen Source	Fermentation Time (h)	Fermentation pH	Mineral Salt	Fermentation Temperature (℃)	Enzyme Activity	References
*Pseudomonas* sp. TKU015	1% Shrimp shell waste	1% Shrimp shell wastes	48	7	0.1% K_2_HPO_4_ 0.05% MgSO_4_·7H_2_O	30	2.3 FU/mL (fibrin as a substrate)	[16]
*Bacillus subtilis* Natto B-12	2% Maltose	2% Wheat	60	7	0.5% NaCl, 0.1% KH_2_PO_4_, 0.4% K_2_HPO_4_, 0.05% MgSO_4_·7H_2_O,	30	903 IU/mL (fibrin as a substrate)	[17]
*Bacillus subtilis* LD-8547	5% Rice powder	4% Soybean powder 0.5% NH_4_NO_3_	72	7	0.5% NH_4_NO_3_, 0.01% CaC1_2_, 0.7% MgSO_4_, 0.4% K_2_HPO_4_, 0.2% KH_2_PO_4_	35	3980 U/mL (fibrin as a substrate)	[20]
*Bacillus subtilis* TKU007	1.5% Shrimp shell powder	-	48	7	0.1% K_2_HPO_4_, 0.05% MgSO_4_	30	0.35 U/mL (casein as a substrate)	[30]
*Bacillus subtilis* RJAS19	1% Lactose	0.5% Peptone	72	9	-	50	-	[28]
*Bacillus subtilis MX-6*	1% Peptone	0.3% Beef extract	72	7.2–7.4	0.04% CaCl_2_, 0.04% MgSO_4_, 0.5% NaCl	37	-	[50]
*Bacillus subtilis* ICTF-1	3% Maltose	0.5% Soy peptone, 1.5% Yeast extract	48	-	0.15% K_2_HPO_4_·3H_2_O, 0.125% MgSO_4_·7H_2_O, 0.025% CaCl_2_·2H_2_O, 1% NaCl	37	8645 U/mg (fibrin as a substrate)	[36]
*Bacillus amyloliquefaciens* DC-4	2% Dextrin	0.15% Yeast extract 2% fibrin, 0.5% Peptone	72	7.2	0.4% K_2_HPO_4_, 0.04% NaH_2_PO_4_, 0.3% CaCO_3_	37	407 U/mg (fibrin as a substrate)	[19]
*Bacillus subtilis* DC33	0.2% Galactose	1.4% Tryptone	72	7.2	0.1% KH_2_PO_4_, 0.2% Na_2_HPO_4_, 0.02% MgSO_4_, 0.3% CaCO_3_, 0.001% FeCl_3_, 0.001% LiSO_4_	37	447.4 U/mg (fibrin as a substrate)	[21]

The neutral pH of the fermentation broth is capable of improving the relative metabolic efficiency of nattokinase fermentation strains, while the rotating speed of the shaker and the volume of the liquid medium indirectly affect the fermentation yield of nattokinase by changing the concentration of dissolved oxygen in the fermentation broth. Low content of dissolved oxygen in the fermentation medium will hinder the growth of *Bacillus subtilis* and the synthesis of nattokinase, and excessive dissolved oxygen will lead to bacterial oxygen poisoning, which will also harm the fermentation of nattokinase. Therefore, an appropriate oxygen level is a necessary condition for the efficient liquid fermentation of nattokinase [51]. During the liquid fermentation of nattokinase, acetoin (an important food spice) is also produced; this method of obtaining multiple beneficial products through one fermentation is also considered to be an effective method to reduce the production cost of nattokinase [52].

In addition to the traditional sources of fermentation raw materials, exploring lower-cost organic substances and even industrial wastes as fermentation raw materials is also a research hotspot for the optimization of the nattokinase liquid fermentation process. It is becoming possible to increase the yield of nattokinase fermentation while reducing production costs and environmental pollution. Tapioca starch is a powder obtained by dehydration and drying of cassava after starch extraction, which has an improved cost advantage compared to traditional fermented carbon sources. It has been reported that the highest enzyme activity of 3787 U/mL of nattokinase can be obtained by combining tapioca starch and soybean as the carbon source and nitrogen source of *Bacillus subtilis* D21-8 strain for fermenting nattokinase [14]. In addition to tapioca flour, cheese whey can also be used as an ideal raw material for nattokinase fermentation; together with yeast extract as a nattokinase fermentation medium, it can not only improve the yield of enzyme fermentation, but also reduce the cost of fermentation broth by nearly half [53]. Li used tofu processing wastewater to replace the nitrogen source in the nattokinase fermentation medium, which reduced production costs and solved the problem of environmental pollution caused by wastewater. The experiment confirmed that the enzyme activity of nattokinase produced by *Bacillus subtilis* 13,932 fermentation increased by 19.25% after optimizing the medium and culture parameters; the subsequent fermentation volume amplification experiment (100 L) showed that the activity of nattokinase was increased by 47.89% [54].

The liquid fermentation method of nattokinase overcomes several shortcomings of the solid-state fermentation method, such as low water content in the medium, poor fluidity, difficult process detection, etc., and the use of industrial waste can solve the cost and environmental pollution problems of nattokinase fermentation. Therefore, this method has gradually become the main nattokinase production method. However, the nattokinase synthesized by liquid fermentation is still difficult to separate and purify, and a large amount of waste will be generated during the fermentation process; thus, it still cannot completely replace the solid-state fermentation process. Therefore, how to solve the problems of product purification and environmental pollution in the process of synthesizing nattokinase by liquid fermentation still needs in-depth research.

## 5. Enzymatic Activity Determination and Enzymatic Properties of Nattokinase

### 5.1. Determination of Fibrinolytic Activity of Nattokinase

Since Nakamura first obtained the nattokinase gene *apr*N, the enzymatic properties of nattokinase from different microbial sources have been studied in detail [37]. The methods for determination of nattokinase fibrinolytic activity and the advantages and disadvantages of each method are shown in Table 3. However, there is no standard method for the determination of nattokinase activity. The most commonly used assays for nattokinase activity include the fibrin plate method, the fibrinolysis method and the tetrapeptide substrate method. The fibrin plate method measures the enzymatic activity of nattokinase by adding nattokinase to agarose plates containing thrombi formed by the interaction of thrombin and fibrinogen to form a clear circle. Although the fibrin plate method is intuitive and simple and able to measure multiple samples at the same time, it has high cost, large detection error and low sensitivity [55]. The principle of the fibrinolysis method is to use urokinase or plasmin as a standard to measure the fibrinolysis time of nattokinase and to calculate the enzyme activity by plotting the logarithm of the concentration of nattokinase and the fibrinolysis time. Although this method has high resolution and short measurement time, it is not suitable for the activity determination of pure enzymes, and it is difficult to measure multiple samples [56]. The principle of the tetrapeptide substrate method is achieved by measuring the absorbance change at 410 nm of the nitroaniline generated after hydrolysis of the tetrapeptide substrate N-Succinyl-L-alanyl-L-alanyl-L-prolyl-L-phenylalanine 4-nitroanilide (N-Succinyl-Ala-Ala-Pro-Phe-pNA) by nattokinase. The tetrapeptide substrate method is simple and sensitive and can rapidly determine the activity of the enzyme, especially the kinetic parameters of the enzyme; however, this method cannot fully express its fibrinolytic activity [44]. Therefore, which method to choose to measure the enzymatic activity of nattokinase depends on the purity of the nattokinase and the type of parameters to be measured.

**Table 3:** Methods of nattokinase activity determination.

Method	Principle	Feature	Reference
Fibrin plate method	Nattokinase hydrolyzes fibrin on the plate to form a hydrolysis circle	The accuracy is not high, only used for preliminary characterization of enzyme activity	[55]
Fibrinolysis method	A standard curve was established using the time taken for urokinase to dissolve a certain amount of fibrin with different activity units.	Simple with limited precision	[56]
Tetrapeptide substrate method	Hydrolysis of Suc-Ala-Ala-Pro-Phe-pNA by nattokinase to produce chromogenic nitroaniline	Simple and fast, high sensitivity; only suitable for pure enzymes	[57]
HPLC method	The product of nattokinase hydrolysis of peptide A (LKRLKRFLKRLK) was detected by a diode array detector and an Eclipse XDB-C18 column.	The operation process is complicated	[58]
Milk plate method	Nattokinase hydrolyzes casein on the plate to form a hydrolysis circle	Simple and fast, used for preliminary characterization of nattokinase activity	[59]
Serum plate method	Nattokinase hydrolyzes fibrin in serum to form hydrolysis circle	Low cost, convenient and quick; easily affected by the quality of the prepared plate.	[60]
Enzyme-linked immunosorbent method	Determination of nattokinase activity by specific enzyme-linked adsorption	High sensitivity and specificity; complicated operation and high cost	[61]
UV spectrophotometer method	Determination of the fibrinolytic activity of nattokinase by measuring the change in absorbance of nattokinase fibrin hydrolysate at 275 nm	The operation process is complicated	[62]

### 5.2. Biochemical Properties of Nattokinase

Table 4 summarizes the enzymatic properties of some of the discovered nattokinases. In general, the molecular weights of nattokinases are between 24–35 kDa. Nattokinase could be stable in the pH range of 6.0–12.0, and rapidly inactivated below pH 5; hence, it is a neutral or weak alkaline enzyme. The poor acid stability of nattokinase results in a rapid loss of activity after oral administration [63]. Compared with fasting administration, nattokinase mixed with boiled meat, rice, and wheat extracts and gastric mucin improved the acid stability of nattokinase in the gastrointestinal tract, which may be related to the reduced contact between nattokinase and gastric juice [64]. The best enzymatic activity of nattokinase can be obtained in the range of 40–65 °C [16,17,28]. Nattokinase showed strong stability under a low-temperature environment; it still retained more than 95% of its enzymatic activity after repeated freezing and thawing five times [4]. Metal ions have different effects on nattokinase. In general, Co^2+^, Mg^2+^ and Ca^2+^ can enhance the activity of nattokinase [29,65]; Mn^2+^ and Ag^+^ have the ability to inhibit the activity of nattokinase to varying degrees [21,66]; Fe^2+^, Zn^2+^, Ba^2+^, Cu^2+^ and Al^3+^ have different effects on different nattokinases, and it is speculated that this phenomenon may be caused by different nattokinase strains and environmental sources [17,26]. Phenylmethanesulfonyl fluoride (PMSF) can completely inhibit the activity of nattokinase, and a certain concentration of phenylmethylsulfonyl chloride, diethylpropyl fluorophosphate and trichlorfon can also completely inhibit its activity [64]. In terms of substrate specificity, most nattokinases can use the synthetic tetrapeptide N-succinyl-Ala-Ala-Pro-Phe-pNA as a substrate to release p-nitroaniline; thus, compared with the agarose-fibrin plate method, the tetrapeptide substrate method is also often used to characterize the enzymatic properties of nattokinase [40,43].

**Table 4:** Enzymatic properties of nattokinase from different microorganisms.

Strain	MW (kDa)	Optimum pH	Optimum Temperature (°C)	Substrate Specificity	Specific Inhibitor	Metal Agonist	Metal Inhibitor	References
*Bacillus subtilis* Natto B-12	29	8	40	-	-	Zn^2+^	Al^3+^, Fe^3+^	[17]
*Bacillus subtilis* BK-17	31	-	-	H-D-Val-Leu-Lys-pNA	Chymostatin	-	Zn^2+^	[67]
*Bacillus* sp. *strain* CK 11-4	28.2	10.5	70	H-D-Val-Leu-Lys-rNA	PMSF	-	-	[23]
*Bacillus* sp. strain DJ-4	29	10	40	-	PMSF	-	Cu^2+^, Zn^2+^	[26]
*Bacillus subtilis* IMR-NK1	31.5	7.8	55	N-succinyl-Ala-Ala-Pro-Phe-pNA	PMSF, NBS	-	-	[68]
*Bacillus amyloliquefaciens* DC-4	28	9	48	N-succinyl-Ala-Ala-Pro-Phe-pNA	PMSF	-	-	[19]
*Bacillus subtilis* DC33	30	8	55	N-Succinyl-Ala-Ala-Pro-Phe-pNA	PMSF, SBTI	-	Cu^2+^, Fe^2+^, Sn^2+^, Ag^+^, Ti^2+^	[21]
*Bacillus subtilis* QK02	28	8.5	55	-	PMSF	-	-	[27]
*Bacillus subtilis* LD-8547	30	8	50	-	PMSF	Al^3+^, Zn^2+^	Mn^2+^, Ba^2+^	[20]
*Bacillus subtilis* TKU007	28–30	7	50	-	-	-	-	[30]
*Bacillus subtilis* RJAS19	35	9.5	65	-	-	-	-	[28]
*Bacillus subtilis* YJ1	27.5	8.5	50	-	PMSF	Co^2+^, Ba^2+^	Fe^3+^, Hg^2+^, Cu^2+^, Zn^2+^	[29]
*Bacillus subtilis* TKU007	28–30	8	50	-	PMSF	-	Ca^2+^, Cu^2+^	[30]
*Pseudomonas* sp. TKU015	21–24	7	50	N-benzoyl-Val-Gly-Arg-pNA and D-Val-Leu-Lys-pNA	PMSF	Fe^2+^	Fe^2+^	[16]
*Pseudomonas aeruginosa* CMSS	27	5	45	-	PMSF	Mn^2+^	Ba^2+^, Mg^2+^	[15]
*Bacillus ereus* VITSDVM3	-	-	-	-	-	-	Cu^2+^, Ca^2+^	[25]
*Bacillus subtilis* WRL101	29	11	47	Met-Suc-Arg-Pro-Tyr-pNA(S-2586)	PMSF	Mg^2+^	Zn^2+^, Cu^2+^	[24]
*Bacillus subtilis* VTCC-DVN-12-01	27.7	9	65	-	SDS	Mg^2+^	-	[65]
*Bacillus subtilis* JNFE0126	-	7	40	-	-	-	-	[18]
*Bacillus subtilis* VITMS 2	29	7	30	N-Succinyl-Ala-Ala-Pro-Phe-pNA (S7338)	PMSF, EDTA	Ca^2+^, Zn^2+^, Co^3+^	Hg^2+^, Cu^2+^	[34]
*Bacillus subtilis* K2	26	-	-	-	-	-	-	[35]
*Bacillus subtilis* ICTF-1	28	9	50	-	PMSF	Zn^2+^, Fe^2+^, Hg^2+^	-	[36]

PMSF, Phenylmethylsulfonyl fluoride; NBS, N-Bromosuccinimide; SBTI, Trypsin inhibitor; SDS, Sodium dodecyl sulfate; EDTA, Ethylene diamine tetraacetic acid.

## 6. Molecular Modification and Expression of Nattokinase

### 6.1. Modification of Fibrinolytic Activity and Stability of Nattokinase

Although many kinds of nattokinase from microorganisms have been found and their enzymatic properties have been characterized, the fibrinolytic activity of nattokinase is still insufficient, which limits the clinical application of this enzyme. To solve the above problems, in recent years, many studies have focused on the structure of nattokinase protein by excavating the key regions or sites affecting the fibrinolytic activity of nattokinase, and on the molecular modification of these regions and sites to improve the fibrinolytic activity of nattokinase, which was summarized in Table 5. Directed evolution simulates the Darwinian evolution process and uses random mutation combined with high-throughput screening to screen out proteins with desired characteristics. Y. Cai et al. screened a nattokinase mutant with 16 mutations to increase the fibrinolytic efficiency by 2.3 times through directed evolution and speculated that the mutant increased the contact efficiency of the enzymatic function by changing the surface conformation of the substrate-binding pocket [69]. Compared with directed evolution, rational analysis based on protein structure can reduce the capacity of mutation library, thereby reducing the intensity of screening work and improving efficiency. Kapoor conducted comparative genomic analysis and biochemical molecular analysis of five different *Bacillus subtilis* derived nattokinases and identified three key residues (Thr130, Asp140, and Tyr217) that are crucial to stabilizing the Michaelis constant (K_m_) of nattokinase. The deletion of any of the three key residues may affect the K_m_ of nattokinase [70]. By site-directed mutagenesis of the S3 site of nattokinase, Wu et al. found that residues Gly100, Ser101, and Lys126 in this region are critical for nattokinase activity; replacing Gly100 of nattokinase with residues containing bulky or positively charged side chains greatly reduced the binding force and catalytic activity of the substrate, while S101L, S101F, and S101W significantly enhanced the activity of nattokinase. Mutation of Leu126 may impair the structure of the active cleft of nattokinase, thereby reducing enzyme activity [71]. By pairing the primary sequence of subtilisin Carlsberg (a homologous protein with better protein hydrolase activity) with the primary sequence of nattokinase, Weng et al. found that the fibrinolytic efficiency of nattokinase mutant I31L increased by 2.3 times, which is speculated to be due to the expansion of the active site sparse pocket of nattokinase [57]. Recently, Liu et al. successfully found the nattokinase mutant Q59E with enhanced fibrinolytic enzyme activity by combining the protein surface charge process, sequence pair, and contribution-based method, which improved the thrombolytic effect of nattokinase in vivo [72].

**Table 5:** Enzyme engineering to improve the enzymatic properties of nattokinase.

Strains	Molecular Modification Strategy	Enzyme Activity Assay Method	Effect	References
*Bacillus subtilis* var. natto	Mutation based on the literature	Tetrapeptide substrate method	The I31L mutant increased catalytic efficiency	[57]
*Bacillus subtilis* var. natto	Mutation based on the literature	Tetrapeptide substrate method	The M222A/I31L mutant increased oxidative stability	[57]
*Bacillus subtilis* QK02	Surface charge engineering, sequence alignment, and mutation based on the literature	Tetrapeptide substrate method	The Q59E mutant increased specific activity	[72]
*Bacillus subtilis* QK02	Surface charge engineering, sequence alignment, and mutation based on the literature	Tetrapeptide substrate method	The S78T mutant improved acid stability	[72]
*Bacillus subtilis* QK02	Surface charge engineering, sequence alignment, and mutation based on the literature	Tetrapeptide substrate method	The Y217K mutant enhanced acid and thermal stability	[72]
*Bacillus subtilis* QK02	Surface charge engineering, sequence alignment, and mutation based on the literature	Tetrapeptide substrate method	The N218D mutant improved thermal stability	[72]
*Bacillus subtilis* QK02	Surface charge engineering, sequence alignment, and mutation based on the literature	Tetrapeptide substrate method	The S78T/Y217K mutant improved acid stability	[72]
*Bacillus subtilis* var. natto strain AS 1.107	Non-oxidative mutation of amino acid residues surrounding the catalytic residue Ser221	Fibrin plate method and tetrapeptide substrate method	The T220S mutant increased oxidative stability	[43]
*Bacillus subtilis* var. natto strain AS 1.107	Non-oxidative mutation of amino acid residues surrounding the catalytic residue Ser221	Fibrin plate method and tetrapeptide substrate method	The M222A mutant increased oxidative stability	[43]
*Bacillus licheniformis* WX-02	Deletion of protease genes and construction of high-efficiency expression system of nattokinase (PHY300PLK + P43 + SsacC + aprN + TamyL)	UV spectrophotometer method	Improved the synthesis efficiency of nattokinase in *Bacillus licheniformis* WX-02	[73]
*Bacillus licheniformis* DW-02	Manipulation of signal peptides and signal peptidases (signal peptide of AprE and signal peptidase SipV)	UV spectrophotometer method	Enhanced secretion efficiency of nattokinase in *Bacillus licheniformis*	[74]
*Bacillus subtilis* WB800	Tandem promoter (PHpaII-PHpaII-PP43)	Fibrin plate method and UV spectrophotometer method	Improved the synthesis efficiency of nattokinase in *Bacillus subtilis* WB800	[75]
*Bacillus subtilis* 168	Sequence trimming and nucleotide optimization of the conserved region of the promoter PsrfA	HPLC method	High-efficiency self-inducible expression of nattokinase in *Bacillus subtilis*	[58]
*strain Bacillus licheniformis* DW2	An optimized single-stranded Shine–Dalgarno (SD) sequence was inserted into the hairpin loop for better ribosome recognition and recruitment.	Milk plate method	Increased the fermentation yield of nattokinase in *Bacillus licheniformis*	[59]
*Bacillus subtilis* WB 800	Deletion of protease genes	Tetrapeptide substrate method	Increased the fermentation yield of nattokinase in *Bacillus subtilis* WB 800	[65]

The unsuitable pH value or relatively narrow optimal pH range of enzyme proteins is one of the key factors limiting the industrial production and pharmaceutical application of enzyme proteins. Although there have been relevant studies on nattokinase nano-controlled release materials, molecular modification based on the structure of nattokinase can more effectively control the production cost, which is beneficial to the industrial production and clinical application of nattokinase products [63,76]. However, there are relatively few studies on the molecular modification of the acid stability of nattokinase (Table 5). Recently, Liu et al. mutated asparagine and glutamine on the surface of nattokinase to aspartic acid and glutamic acid by simulating the process of protein deamidation and obtained the S78T/Y217K nattokinase double mutation. The residual enzyme activity of the body was increased by 2.6 times under the condition of pH 4 [72]. At present, the rational design based on the enzyme structure is an effective method to improve the acid–base stability of nattokinase, including the modification of the key amino acid residues in the catalytic active center (or the amino acid residues in its vicinity) and the surface amino acid modification. The transformation of the key amino acid residues in the catalytic active center is more likely to obtain nattokinase with improved acid–base stability, but it is still impossible to ignore that the replacement of such key amino acids is likely to cause confusion in the center area of the active structure, resulting in a decrease in the activity of the enzyme molecule and a change in the substrate specificity [77]; The modification of the amino acid residues on the surface of the enzyme is able to change the optimal pH of the enzyme by changing the surface electrostatic potential of the enzyme or the interaction between the amino acid residues of the enzyme, but the screening workload of this method is relatively large [78]. We believe that the application of the above method can further improve the acid stability of nattokinase and improve the stability of nattokinase in the gastrointestinal tract.

Proteins containing residues methionine, cysteine, or tryptophan in or around the active site are sensitive to chemical oxidation, which easily leads to protein inactivation or denaturation. Aiming at the defect of unstable oxidative stability of nattokinase, by screening the amino acid residues around the catalytic center residue Ser221, Weng designed T220S and M222A mutants with improved oxidation stability: Ser220 and Ala222 affect the oxidative stability of nattokinase by forming hydrogen bonds with Asn 155 and changing the spatial structure of nattokinase, respectively (Table 5) [43].

### 6.2. Construction of Nattokinase Expression System

The use of genetically engineered bacteria to express nattokinase has been proven to be an important way to increase the yield of nattokinase. At present, nattokinase has been expressed in *Escherichia coli* [79], *Lactobacillus* [80], *Bacillus* [65] and *Pichia pastoris* [81]. *Escherichia coli* is the most widely studied host for nattokinase expression. However, the expression of nattokinase in *Escherichia coli* results in mostly inactive inclusion bodies [82]. Although the soluble expression of nattokinase in *Escherichia coli* has been reported, the fibrinolytic activity of recombinant nattokinase is still lower than that of natural nattokinase synthesized by *Bacillus*. Therefore, how to realize the soluble expression of nattokinase encoding gene in *Escherichia coli* has become an urgent problem to be solved [79]. Compared with the problem of insoluble expression of nattokinase in *Escherichia coli*, *Lactococcus lactis* [80], *Lactobacillus bulgaricus* [83] and *Pichia pastoris* were used as hosts to express soluble recombinant nattokinase with thrombolytic activity, showing the great potential of nattokinase in the food and pharmaceutical industries [80,81,83].

At present, many studies have confirmed that the synthesis efficiency and fibrinolytic activity of nattokinase in *Bacillus* can be greatly improved by optimizing the regulatory elements upstream of the open reading frame of nattokinase. For example, replacing the promoter and signal peptide upstream of the nattokinase encoding gene was confirmed to improve the expression efficiency and secretion rate of nattokinase during *Bacillus* fermentation, respectively. Cai et al. replaced the signal peptide upstream of the nattokinase-encoding gene, heterologously expressed in *Bacillus licheniformis* with nattokinase, and successfully improved the secretion efficiency of nattokinase [74]. By simultaneously adjusting the promoter gene (P43), signal peptide gene (SsacC), and terminator gene (α-amylase) for expressing nattokinase, Wei et al. successfully achieved high-level expression of recombinant nattokinase [73]. Liu et al. transformed the recombinant plasmid (pMA0911) containing the tandem promoter (PHpaII-PHpaII-PP43) and the nattokinase encoding gene into recombinant *Bacillus subtilis* WB800, and the activity of nattokinase reached 816.7 ± 30.0 FU/mL after fermentation in a 5 L fermenter [75]. Cheng et al. optimized the promoter upstream of the gene encoding nattokinase, and the optimized promoter significantly improved the recombinant expression of nattokinase in *Bacillus subtilis* 168 [58]. In addition, Xiao et al. developed a portable 5′-UTR sequence for enhanced protein production from the industrial strain *Bacillus licheniformis* DW2. This 5′-UTR effectively enhanced green fluorescent protein expression by approximately 50-fold and exhibited good adaptation to nattokinase [59]. Knockout of the expression of nattokinase-degrading protease was also confirmed to promote the synthesis of nattokinase in *Bacillus*. Wei et al. improved the fermentation yield of nattokinase by knocking out the protease that may hydrolyze nattokinase [73]. Nguyen et al. constructed a *Bacillus subtilis* WB 800 strain lacking eight extracellular protease genes (nprE, aprE, epr, bpr, mpr::ble, nprB::bsr, vpr, wprA::hyg), which fermented nattokinase with a yield of 600 mg/L [65].

Different expression systems have their own advantages and disadvantages due to their own characteristics. The *Escherichia coli* expression system has relatively high expression levels and is easy to purify, but the expression products are mostly inactive or extremely low-activity inclusion bodies, which require renaturation of inclusion bodies to obtain functional enzymes; *Bacillus subtilis* had the best expression activity, but the existence of impurity proteins in the fermentation broth increased the difficulty of purification; the *Pichia pastoris* expression system can obtain high-purity nattokinase, but the enzyme activity is low and the expression amount is relatively small. How to further improve the recombinant expression efficiency of nattokinase through site-directed mutagenesis of nattokinase, co-expression of nattokinase propeptide, optimization of regulatory elements, selection of protease-deficient host cells, and optimization of the purification process of recombinant nattokinase will be the focuses of future research.

## 7. Clinical Application of Nattokinase

### 7.1. Study on Thrombolytic Effect of Nattokinase

**Figure 3:**
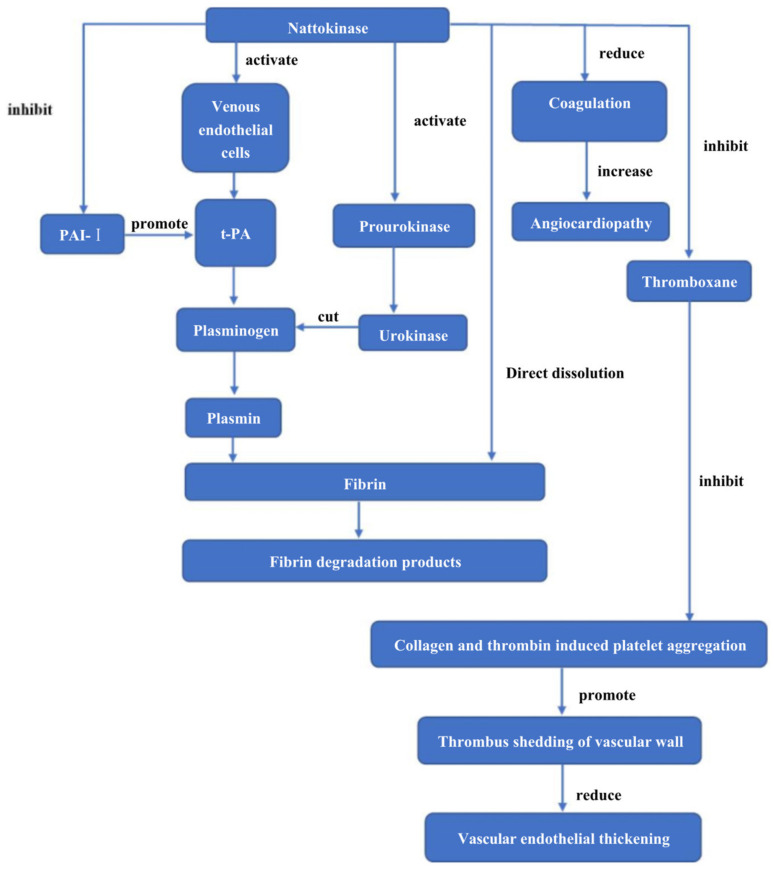
Thrombolytic mechanism of nattokinase.

The traditional antithrombotic drugs have a poor thrombolytic effect, bleeding tendency, and single-mode of administration, while the high thrombolytic activity, low side effects, and oral administration of nattokinase make it a potential thrombolytic alternative drug. Fujita et al. first discovered the thrombolytic effect of nattokinase in acetic acid-induced arterial thrombosis rats. They found that the thrombolytic activity of nattokinase was much higher than that of urokinase [13]. As nattokinase is a new thrombolytic drug with great application potential, its thrombolytic mechanism has been studied. Nattokinase has dual functions of direct thrombolysis and indirect thrombolysis. In terms of the direct thrombolytic mechanism, nattokinase can hydrolyze the thrombus into amino acids and small peptides and then directly achieve the thrombolytic effect. The indirect thrombolytic mechanism of nattokinase is more diverse (Figure 3). (1) Nattokinase activates the conversion of prourokinase to urokinase, together with tissue-type plasminogen to activate plasminogen to generate plasmin [84]. (2) Nattokinase promotes plasminogen transformation by stimulating vascular endothelial cells to produce tissue plasminogen activator (t-PA) [85]. (3) Nattokinase also has the ability to degrade inactivated plasminogen activator type I inhibitor, promote the synthesis of more t-PA and urokinase, and indirectly promote thrombolysis [86]. (4) Nattokinase administration is capable of reducing the concentration of plasma coagulation factor VII and coagulation factor VIII that can increase the risk of cardiovascular diseases by triggering the coagulation cascade reaction [7]. (5) Nattokinase prevents the formation of thromboxane and significantly inhibits platelet aggregation mediated by collagen and thrombin, thereby delaying the formation of thrombi after oxidative arterial wall injury, and promoting the detachment of thrombi from blood vessel walls, so as to reduce endothelial damage and vascular endothelial thickening caused by thrombi [87,88]. A recent study suggested that nattokinase inhibited the activation of the coagulation system induced by inflammation by downregulating the production of reactive oxygen species (ROS) and the activation of nuclear factor-κB (NF-κB), thereby reducing the rate of thrombosis in a mouse model of glomerular thrombus [12].

In addition to its use in the treatment of thrombotic diseases, nattokinase has also been shown to prevent thrombosis. Compared with traditional drugs such as aspirin, nattokinase can not only effectively prevent the formation of blood clots, but also avoid side effects such as bleeding tendency and gastric ulcers [89]. Kamiya et al. demonstrated that rats prophylactically treated with nattokinase had smaller infarcted areas following tail injection of carrageenan than untreated control rats [90]. The effect of nattokinase on alleviating atherosclerosis remains controversial. Oral administration of nattokinase can reduce carotid plaque areas and common carotid artery media thickness in hyperlipidemic patients with lateral carotid atherosclerotic plaques [91]. However, a 3-year study in 265 subjects without cardiovascular disease found no significant annual changes in carotid intima-media thickness and carotid stiffness between nattokinase-treated and control groups [92].

The mechanism of nattokinase blood absorption after oral administration is still not fully resolved. First, intraduodenal administration of nattokinase at a dose of 80 mg/kg was able to induce fibrinolysis in rat plasma, implying that this dose was too high [13]. Second, preliminary studies on the pharmacokinetics of nattokinase are conflicting. Ero et al. claimed that the concentration of nattokinase peaked at 13.3 h after oral administration [93], while Kurosawa believed that the thrombolytic activity of nattokinase peaked at 2 to 4 h after oral administration [94]. Finally, due to the large molecular weight of nattokinase, some studies consider that nattokinase cannot be absorbed through the gastrointestinal tract [95,96]. In addition to the lack of pharmacokinetic studies of nattokinase, the safety of nattokinase also needs further study. Recent studies have shown that natto mucus can cause allergic reactions and symptoms of chronic urticaria by increasing CD203c levels in basophils [97]. The γ-PGA in natto mucus is considered to be an important cause of delayed allergic reaction and chronic urticaria symptoms [98,99].

### 7.2. Antihypertensive Effect of Nattokinase

Hypertension can directly increase the probability of stroke and myocardial infarction, and it is considered to be one of the most important risk factors for cardiovascular diseases. Antihypertensive drugs such as angiotensin-converting enzyme inhibitors, thiazide diuretics, and angiotensin II receptor blockers must be used in consideration of many factors, including adverse reactions, contraindications, and special populations [100]. Nattokinase has a significant antihypertensive effect and high safety and is expected to be an alternative for the treatment of hypertensive patients.

Oral administration of nattokinase relieves symptoms of hypertension mainly by effectively reducing systolic and diastolic blood pressure. Oral administration of nattokinase can reduce blood pressure in spontaneously hypertensive rats, and the intact nattokinase and its hydrolyzed fragments can be absorbed from the intestinal tract [101]. Human experiments also confirmed the antihypertensive effect of nattokinase. Kim et al. found that nattokinase reduced systolic blood pressure (−5.55 mmHg) and diastolic blood pressure (−2.84 mmHg) in subjects with pre- and stage 1 hypertension after oral administration of nattokinase for 8 weeks [102]. In a clinical study, Gitte et al. found that both systolic and diastolic blood pressure were reduced in the nattokinase group compared to the placebo group after 8 weeks, and interestingly, the antihypertensive effect of oral nattokinase was higher in men than in women [8]. The pharmacological mechanism of nattokinase antihypertensive remains to be studied. It is now clear that nattokinase can reduce blood pressure by inhibiting the secretion of angiotensin-converting enzyme I and angiotensin II, and interact with fibrinogen in plasma to reduce blood viscosity, thereby lowering blood pressure [103].

### 7.3. Potential Applications of Nattokinase in Other Diseases

In addition to the research of nattokinase in the fields of thrombosis and hypertension treatment, nattokinase can also be used as a drug or adjuvant to treat diseases such as cancer [11], Alzheimer’s disease (AD) [104], retinopathy [10], hyperlipidemia [105] and oral mucositis [106]. Nattokinase has broad prospects in the field of cancer treatment and is expected to become an effective means of cancer treatment in the future. Liver cancer has a very high incidence worldwide. Administration of nattokinase was shown to inhibit the expression of transcription factors CD31, FOXM1, CD44, and vimentin that regulate tumor proliferation, survival, and drug resistance in a mouse model of liver cancer, thereby reducing the tumor growth rate [11]. Cancer-associated thrombosis (CAT) is associated with poor prognosis in tumor therapy, and thrombotic complications are the second leading cause of death in colon cancer patients [107]. CAT can not only promote the invasion and infiltration of tumor cells but also provide a physical barrier that affects the entry of immune cells and therapeutic drugs into the tumor to thwart the therapeutic effect, especially in advanced tumors with thrombosis [108,109,110]. Therefore, the development of CAT-dissolving drugs and their delivery systems is a promising strategy for the treatment of cancer and thrombotic complications. The ability of nattokinase to dissolve CAT makes it an adjunct to cancer therapy [111]. A conjugate of polyethylene glycol-polyglutamic acid peptide dendrimers and nattokinase formed by disulfide bonds has good thrombolytic properties in vitro, and its doxorubicin-loaded polymer conjugate had good endocytic ability and anticancer effects on HCT116 cells [112]. The nattokinase–PAS complex formed by the electrostatic interaction between polysialic acid (PAS) and nattokinase can transfer nattokinase into CAT for thrombolysis. The study confirmed that the combined use of nattokinase-PAS and doxorubicin liposome significantly improved the antitumor effect of doxorubicin plastid [113].

AD is a neurodegenerative disease characterized by the presence of amyloid plaques in the form of fibrillar proteins in the brain, and nattokinase has a strong ability to hydrolyze amyloid [114]. In an aluminum chloride-induced AD rat model, oral administration of nattokinase not only reduced acetylcholinesterase (AchE) activity, but transformed growth factor-beta (TGF-β), Fas, and interleukin-6 (IL-6) content in the AD pathway and also restored the expression of metalloproteinase domain (9ADAM9) and metalloproteinase domain (10ADAM10) genes [115]. Furthermore, treatment with nattokinase suppressed AChE activity and oxidative stress in a mouse model of colchicine-induced learning and memory impairment, thereby improving neurobehavior [104]. Conjugated poly(lactic-co-glycolic acid) (PLGA)-encapsulated nattokinase with Tet1 peptide as a nanoformulation of nattokinase exhibits affinity for neurons and reduces amyloid aggregation, so it may also be a potential drug for the treatment of AD [116].

Diseases such as retinopathy of prematurity, diabetic retinopathy, and retinal vein occlusion all cause irreversible visual impairment and blindness through the formation of pathological neovascularization. Nattokinase attenuated neovascularization in the retinas of mice with oxygen retinopathy but activated the Nrf2/HO-1 pathway to ameliorate ischemic retinopathy [10]. In addition, nattokinase has been proved to induce posterior vitreous detachment, and it is a useful drug as a vitreous cleavage enzyme that has application prospects in the treatment of proliferative vitreoretinal diseases [117].

In addition to its antithrombotic function, nattokinase has been reported to lower blood pressure, arteriosclerosis, coronary heart disease (such as angina pectoris), stroke and peripheral vascular disease. However, the molecular mechanism of nattokinase in the treatment of these diseases remains to be explored, and the dosage and toxicity of administration are still unclear. The solution of these problems can establish a theoretical basis and technical support for us to better use nattokinase.

## 8. Prospects

*Bacillus* nattokinase is a potential thrombolytic drug with a powerful thrombolytic effect, diverse mechanisms, and safety, and is becoming a focus of cardiovascular drug research. Although current research on the pharmacodynamics and pharmacology of nattokinase has been completed, there are still many problems in the application of nattokinase in the fields of medicine and functional food. First of all, the product quality evaluation of nattokinase still needs to be improved; the current quality evaluation of nattokinase products mainly relies on the determination of enzyme activity, which is not comprehensive. The activity evaluation of nattokinase combined with its stability evaluation under different temperatures, pH, and other influencing factors will be more conducive to the development of nattokinase health foods and thrombolytic drugs. Secondly, the physicochemical properties of nattokinase are still insufficient for industrial production and application: as a macromolecular compound, commercially available nattokinase has relatively insufficient activity and is very sensitive to acid, high temperature, and oxides. Therefore, it is easy for the activity of nattokinase to be lost during production, preservation, and oral administration, which limits its clinical application. Effective means to solve the stability defects of nattokinase include but are not limited to the following: (1) screen nattokinase strains with strong activity and stability, and explore fermentation processes to improve the secretion efficiency of nattokinase; (2) stabilize nattokinase by physical embedding, nanoparticle binding, temperature, and pH control, and adjustment of coexisting substances; (3) use modern genetic engineering technology and mutation breeding technology to improve the activity and stability of nattokinase. The combination of drugs is a common method for the clinical treatment of diseases. Although reports on the combination of nattokinase with other drugs are lacking, research on the combination of nattokinase with other cardiovascular disease drugs may provide a new approach to the treatment of cardiovascular disease.

## Data Availability

Not applicable.
